# Noninfluenza Vaccination Coverage Among Adults — United States, 2011

**Published:** 2013-02-01

**Authors:** Walter W. Williams, Peng-Jun Lu, Stacie Greby, Carolyn B. Bridges, Faruque Ahmed, Jennifer L. Liang, Tamara Pilishvili, Craig Hales

**Affiliations:** Immunization Services Div; Div of Bacterial Diseases; Div of Viral Diseases, National Center for Immunization and Respiratory Diseases, CDC

Vaccinations are recommended throughout life to prevent vaccine-preventable diseases and their sequelae. Adult vaccination coverage, however, remains low for most routinely recommended vaccines (1) and well below *Healthy People 2020* targets.* In October 2012, the Advisory Committee on Immunization Practices (ACIP) approved the adult immunization schedule for 2013 (2). Apart from influenza vaccination, which is now recommended for all adults, other vaccines recommended for adults target different populations based on age, certain medical conditions, behavioral risk factors (e.g., injection drug use), occupation, travel, and other indications (2). To assess adult (aged ≥19 years) vaccination coverage for select vaccines, CDC analyzed data from the 2011 National Health Interview Survey (NHIS). This report summarizes the results of that analysis for pneumococcal vaccine, tetanus toxoid–containing vaccines (including tetanus and diphtheria toxoid [Td] with acellular pertussis vaccine [Tdap]), and hepatitis A, hepatitis B, herpes zoster (shingles), and human papillomavirus (HPV) vaccines, by selected characteristics (age, race/ethnicity,† and vaccination target criteria). Influenza vaccination coverage estimates for the 2011–12 influenza season have been published separately (3). Compared with 2010 (1), the data indicate modest increases in Tdap vaccination among persons aged 19–64 years and HPV vaccination among women, but only little improvement in coverage for the other vaccines among adults in the United States. Coverage for tetanus vaccination (with any tetanus toxoid–containing vaccine) during the past 10 years was unchanged. Substantial increases in vaccination coverage are needed to reduce the occurrence of vaccine-preventable diseases among adults. The Community Preventive Services Task Force and other authorities have recommended that health-care providers incorporate vaccination needs assessment, recommendation, and offer of vaccination into routine clinical practice for adult patients (4,5).

NHIS collects information about the health and health care of the noninstitutionalized, civilian population in the United States using nationally representative samples. Interviews are conducted in respondents’ homes by the U.S. Census Bureau for CDC’s National Center for Health Statistics. Questions about receipt of recommended vaccinations for adults are asked of one randomly selected adult within each family in the household. The presence of high-risk conditions,§ as defined by ACIP for each vaccine, was determined by responses to questions in the NHIS (2). The final sample adult component response rate for the 2011 NHIS was 66.3%. Weighted data¶ were used to produce national estimates. Point estimates and estimates of corresponding variances were calculated using statistical software to account for the complex sample design. Statistical significance was defined as p<0.05.

## Pneumococcal Vaccination Coverage

Pneumococcal vaccination coverage among adults aged 19–64 years at high risk was 20.1% overall, a 1.6 percentage point increase from 2010 (Table 1). Coverage among whites aged 19–64 years at high risk was higher (20.1%) compared with Hispanics (18.3%) and Asians (12.0%), but coverage was not significantly different for other racial/ethnic groups. Among adults aged ≥65 years, coverage was 62.3% overall, a 2.6 percentage point increase from 2010. Coverage among whites aged ≥65 years increased from 2010 (by 3.0 percentage points to 66.5%) and was higher compared with Asians (40.3%), Hispanics (43.1%), and blacks (47.6%).

## Tetanus Vaccination Coverage

In 2011, the proportion of adults receiving any tetanus toxoid–containing vaccination (i.e., Td or Tdap) during the past 10 years was 64.5% for adults aged 19–49 years, 63.9% for adults aged 50–64 years, and 54.4% for adults aged ≥65 years (Table 1). The proportion of adults receiving tetanus vaccination during the past 10 years across all age groups did not change compared with 2010 (1). Whites had higher coverage across all age groups compared with Asians, Hispanics, and blacks.

Among adults aged 19–64 years for whom Tdap vaccination specifically could be assessed, Tdap coverage increased compared with 2010 (a 4.3 percentage point increase to 12.5%) (Table 1). Tdap coverage was estimated after excluding from the 25,783 respondents all those without a “yes” or “no” response for tetanus vaccination status in the past 10 years (n = 1,118 [4.3%]) or tetanus vaccination status during 2005–2011 (n = 803 [3.1%]), and those who reported tetanus vaccination during 2005–2011 but were not told (n = 5,501 [21.3%]) or did not know the vaccine type (n = 881 [3.4%]) (Td or Tdap). Among 9,805 respondents who received a tetanus vaccination during 2005–2011, 55.9% reported that they were not informed of the vaccination type, and 8.9% could not recall what type of tetanus vaccination they had received (Table 2). Of the remaining 35.2% of respondents who reported they knew what type of tetanus vaccine they received, 61.1% reported receiving Tdap.

Compared with 2010, Tdap coverage increased among all racial/ethnic groups except Asians. For white and black respondents, coverage increased by 4.7 and 3.6 percentage points, respectively, to 13.8% and 11.0%. For Hispanic respondents, coverage increased by 2.9 percentage points to 7.7% (Table 1). The largest increase occurred among adults aged 19–64 years who indicated a race other than Asian, black, or white, and non-Hispanic ethnicity (a 11.3 percentage point increase to 19.7%). Increases compared with 2010 also occurred among persons with and without household contact with an infant aged <1 year** (a 10.9 percentage point increase to 21.5%, and a 4.0 percentage point increase to 12.1%, respectively). However, reported Tdap coverage among persons aged 19–64 years remained low overall. Whites had higher Tdap coverage (13.8%) compared with blacks (11.0%) and Hispanics (7.7%).

During 2005–2011, Tdap vaccination of health-care personnel (HCP) (26.8%) was 6.5 percentage points higher than the 2010 estimate (Table 3). White HCP had higher Tdap coverage (27.2%) compared with black HCP (21.7%). Compared with 2010, Tdap coverage increased for Hispanic HCP (by 16.3 percentage points to 30.1%) and was similar to that of white HCP.

Among persons aged 19–64 years who received a tetanus vaccination, HCP were more likely to report receipt of Tdap (66.8%) than non-HCP (59.7%) (Table 2).

## Hepatitis A Vaccination Coverage

Compared with 2010, overall hepatitis A vaccination coverage (≥2 doses) increased among adults aged 19–49 years (by 1.8 percentage points to 12.5%) but remained low. Vaccination coverage was higher (20.1%) among adults aged 19–49 years who had traveled outside the United States since 1995 to a country of high or intermediate endemicity than among respondents who had traveled only to countries of low endemicity (8.4%) (Japan, Australia, New Zealand, Canada, and the countries of Europe). Vaccination coverage among adult travelers to highly endemic countries increased by 3.5 percentage points from 2010 to 2011 (Table 1). Coverage was higher for Asians (19.1%) and adults aged 19–49 years who indicated a race other than Asian, black, or white and non-Hispanic ethnicity (21.1%) than for other groups. Coverage among those with chronic liver conditions (17.1%) was similar to the estimate for 2010.

## Hepatitis B Vaccination Coverage

In 2011, information on high-risk status for hepatitis B virus infection was not collected. Overall hepatitis B vaccination coverage (≥3 doses) among all adults aged 19–49 years was 35.9% (2.1 percentage points higher than the 2010 estimate) (Table 1). Vaccination coverage was lower for blacks (33.0%) and Hispanics (28.9%) compared with whites (37.8%). Vaccination coverage for persons with diabetes was 26.9% for those aged 19–59 years and 12.4% for those aged ≥60 years, similar to the estimates for 2010. Overall, hepatitis B vaccination coverage among HCP was 63.8%, similar to the estimate for 2010. Coverage for black HCP (57.1%) and Hispanic HCP (59.4%) was lower compared with white HCP (65.1%), but coverage for Asian HCP (70.4%) was higher than that for white HCP (Table 3).

## Herpes Zoster Vaccination Coverage

In 2011, 15.8% of adults aged ≥60 years reported receiving herpes zoster vaccination to prevent shingles, similar to the estimate for 2010 (Table 1). Whites aged ≥60 years had higher herpes zoster vaccination coverage (17.6%) compared with blacks (7.9%), Hispanics (8.0%), and Asians (14.0%). Coverage for blacks and Hispanics aged ≥60 years increased by more than 3 percentage points compared with herpes zoster vaccination coverage estimates in 2010.

## HPV Vaccination Coverage

In 2011, 29.5% of women aged 19–26 years reported receipt of ≥1 dose of HPV vaccine, an increase from the 20.7% reported for 2010 (Table 1) (1), and a further increase from the 17.1% reported for 2009 (1). Coverage was 43.1% among women aged 19–21 years and 21.5% among those aged 22–26 years. Among women aged 19–26 years, Hispanics had lower coverage (20.2%) compared with whites (32.5%), but coverage across racial/ethnic groups otherwise did not differ. Compared with 2010, receipt of ≥1 dose of HPV vaccine increased among males aged 19–26 years (by 1.5 percentage points to 2.1%). Coverage was 2.8% for males aged 19–21 years and 1.7% for those aged 22–26 years.

### Editorial Note

In 2011, noninfluenza adult vaccination coverage in the United States was similar to 2010, except for modest increases in Tdap vaccination overall and HPV vaccination among women, with little or no improvements in coverage for the other vaccines recommended for adults. Many adults have not received one or more recommended vaccines. Vaccination coverage estimates for the three vaccines in this report that are included in *Healthy People 2020* (pneumococcal, herpes zoster, and hepatitis B [for HCP] vaccines) are well below the respective target levels of 90% for persons aged ≥65 years and 60% for persons aged 18–64 years at high risk (pneumococcal vaccine [objectives IID 13.1 and IID 13.2, respectively]), 30% (herpes zoster vaccine [IID 14]), and 90% (hepatitis vaccine for HCP [IID 15.3]). These data indicate little progress was made in improving adult coverage in the past year and highlight the need for continuing efforts to increase adult vaccination coverage.

Since 2006, ACIP has recommended that adults aged 19–64 years receive a single dose of Tdap to replace a dose of Td for active booster vaccination against tetanus, diphtheria, and pertussis if they received their most recent dose of Td ≥10 years earlier (6). In October 2010, ACIP recommended expanded use of Tdap, indicating that adults aged ≥65 years who have or who anticipate having close contact with an infant aged <1 year, and who previously have not received Tdap, should receive a single dose of Tdap to protect against pertussis and reduce the likelihood of transmission. ACIP also recommended that Tdap, when indicated, be administered regardless of the interval since the most recent tetanus or diphtheria toxoid–containing vaccine was received (6). Information on Tdap vaccination of adults aged ≥65 years was not collected in the 2011 NHIS but is being collected starting in 2012. In February 2012, ACIP recommended that all adults aged ≥19 years who have not yet received a dose of Tdap should receive a single dose regardless of the interval since the most recent tetanus or diphtheria toxoid–containing vaccine was received.†† These recommendations supersede previous Tdap recommendations regarding adults aged ≥65 years. Health-care providers should not miss an opportunity to vaccinate persons aged ≥19 years who have not received Tdap previously.

In June 2012, ACIP recommended routine use of 13-valent pneumococcal conjugate vaccine (PCV13) in series with the 23-valent pneumococcal polysaccharide vaccine (PPSV23) for adults aged ≥19 years with immunocompromising conditions, functional or anatomic asplenia, cerebrospinal fluid leaks, or cochlear implants.§§ Given the high burden of invasive pneumococcal disease caused by serotypes in PPSV23 but not in PCV13, ACIP noted that broader protection might be provided through use of both pneumococcal vaccines. Current ACIP recommendations call for use of PPSV23 in adults aged 19–64 years with chronic conditions that are not immunocompromising, such as chronic heart disease or diabetes, at the time of diagnosis of the high-risk condition (6). All adults are eligible for a dose of PPSV23 at age 65 years, regardless of previous PPSV23 vaccination; however, a minimum interval of 5 years between PPSV23 doses should be maintained. The 2012 NHIS cannot estimate the proportion of pneumococcal vaccinations by type (PCV13 versus PPSV23).

The findings in this report provide baseline estimates of hepatitis B vaccination coverage of adults with diabetes. The ACIP-recommended administration of hepatitis B vaccine to unvaccinated adults with diabetes aged 19–59 years (category A recommendation) or aged ≥60 years (category B recommendation) in December 2011 (6). The recommendations were based on available information about risk for contracting acute hepatitis B among persons with diabetes, morbidity and mortality, available vaccines, age at diagnosis of diabetes, and cost-effectiveness (6).

The percentage of age-eligible females administered HPV vaccine has increased steadily during 2009–2011 but is still low. The largest increase in 2011 (14.9 percentage points) was reported among women aged 19–21 years. This finding might reflect the knowledge, attitude, and practices of the health-care providers of young women (7); the social norms of young women and the perceptions and vaccination intentions of peers (8); or receipt of vaccine when eligible for the Vaccines for Children Program (age <18 years) but aged ≥19 years when interviewed (7). The percentage of age-eligible adult males administered HPV vaccine increased by 1.5 percentage points but remained very low. The ACIP recommendation for routine use of HPV vaccine in females age 11–26 years was made in 2006, whereas use in males aged 11–21 years and males aged 22–26 years at high risk was recommended in October 2011 (6). Thus, coverage levels for males in 2011 would not reflect this new recommendation. The primary target group for HPV vaccine is girls and boys aged 11–12 years.

What is already known on this topic?

During 2008–2010, coverage with routinely recommended vaccinations among U.S. adults aged ≥19 years remained low.

What is added by this report?

Compared with 2010 estimates, modest gains occurred in human papillomavirus vaccination coverage among women aged 19–26 years and in tetanus and diphtheria toxoid with acellular pertussis vaccine (Tdap) vaccination overall and among household contacts of children. Coverage for other vaccines and risk groups increased little, and racial/ethnic disparities persisted for routinely recommended adult vaccines. Coverage for all vaccines for adults remained low.

What are the implications for public health practice?

Despite improvements in vaccination, coverage remains low for most vaccines routinely recommended for adults. Wider use of practices shown to improve adult vaccination is needed, including assessment of patients’ vaccination needs by health-care providers and routine recommendation and offering of needed vaccines to adults, implementing reminder-recall systems, use of standing order programs for vaccination, and assessment of practice-level vaccination rates with feedback to staff members.

The findings in this report are subject to at least five limitations. First, the NHIS sample excludes persons in the military and those residing in institutions, which might result in underestimation or overestimation of vaccination coverage levels. Second, the response rate was 66.3%. A low response rate can result in sampling bias if the nonresponse is unequal among the participants regarding vaccination. Third, the determination of vaccination status and identification of high-risk conditions in NHIS were not validated by medical records. Self-report of vaccination is subject to recall bias and overestimation of rates. However, adult self-reported pneumococcal vaccination status has been shown to be sensitive and specific (9). Fourth, the Tdap estimate is subject to considerable uncertainty. Many respondents were excluded from estimations of Tdap coverage, creating a potential for bias. All respondents who reported a tetanus vaccination during 2005–2011 but were unable to say whether Td or Tdap was used, were excluded. Sensitivity calculations were conducted to assess the magnitude of potential bias. Depending on what proportion of excluded respondents actually received Tdap, actual Tdap coverage could fall within the range of 8.0%–36.4%. Comparisons of Tdap coverage across years within subgroups might be affected by bias resulting from excluding persons who did not report the type of tetanus vaccine they received. Finally, age at vaccination is not known for vaccines adults reported having “ever” received (e.g., HPV and hepatitis B vaccines), so it is not clear for younger adults whether vaccination occurred as an adult or was given as part of a child or adolescent vaccination program.

Vaccination coverage levels among adults are unacceptably low. Substantial improvement in adult vaccination is needed to reduce the health consequences of vaccine-preventable diseases among adults. Successful vaccination programs combine 1) education of potential vaccine recipients and publicity to promote vaccination; 2) increased access to vaccination services in medical and complementary settings, such as workplaces and commercial establishments (e.g., pharmacies); and 3) use of practices shown to improve vaccination coverage, including reminder-recall systems, efforts to remove administrative and financial barriers to vaccination, use of standing order programs for vaccination, and assessment of practice-level vaccination rates with feedback to staff members (5). Health-care provider recommendations for vaccination are associated with patient vaccination (10). Routine assessment of adult patient vaccination needs, recommendation, and offer of needed vaccinations for adults should be incorporated into routine clinical care of adults (4,5). The adult immunization schedule (2), updated annually, provides current recommendations for vaccinating adults and a ready resource for persons who provide health-care services for adults in various settings.

## Figures and Tables

**TABLE 1:** Estimated proportion of adults aged ≥19 years who received selected vaccinations, by age group, high-risk status,* race/ethnicity,† and other selected characteristics — National Health Interview Survey, United States, 2011

Characteristic	No. in sample	%	(95% CI)	Percentage point difference from 2010
**Pneumococcal vaccination, ever** §				
19–64 yrs, high-risk, total	9,056	20.1	(19.1–21.1)	1.6¶
19–64 yrs, high-risk, white	5,510	20.1	(18.9–21.4)	1.1
19–64 yrs, high-risk, black	1,547	22.8	(20.3–25.5)	4.2
19–64 yrs, high-risk, Hispanic	1,365	18.3	(15.8–21.1)**	3.5
19–64 yrs, high-risk, Asian	354	12.0	(8.6–16.6)**	0.5
19–64 yrs, high-risk, other	280	21.7	(16.7–27.7)	−4.4
≥65 yrs, total	6,641	62.3	(60.7–63.8)	2.6¶
≥65 yrs, white	4,739	66.5	(64.8–68.2)	3.0¶
≥65 yrs, black	840	47.6	(43.1–52.2)**	1.8
≥65 yrs, Hispanic	664	43.1	(38.6–47.8)**	4.2
≥65 yrs, Asian	297	40.3	(34.5–46.4)**	−7.9
≥65 yrs, other	101	67.4	(54.1–78.4)	9.0
**Tetanus vaccination, past 10 yrs** ††				
19–49 yrs, total	16,843	64.5	(63.5–65.4)	0.5
19–49 yrs, white	8,889	69.6	(68.4–70.8)	0.3
19–49 yrs, black	2,509	54.8	(52.1–57.4)**	−2.0
19–49 yrs, Hispanic	3,793	56.3	(54.1–58.5)**	1.9
19–49 yrs, Asian	1,223	52.5	(48.9–56.0)**	2.2
19–49 yrs, other	429	69.6	(64.0–74.8)	7.4
50–64 yrs, total	7,822	63.9	(62.4–65.3)	0.5
50–64 yrs, white	4,997	67.7	(66.0–69.4)	0.4
50–64 yrs, black	1,270	54.4	(51.0–57.9)**	1.7
50–64 yrs, Hispanic	1,040	52.6	(48.8–56.4)**	1.7
50–64 yrs, Asian	359	45.1	(38.9–51.4)**	−2.7
50–64 yrs, other	156	67.9	(58.4–76.1)	−0.5
≥65 yrs, total	6,471	54.4	(52.9–56.0)	1.1
≥65 yrs, white	4,612	57.0	(55.2–58.7)	0.6
≥65 yrs, black	809	44.4	(40.0–48.8)**	4.7
≥65 yrs, Hispanic	666	45.1	(40.7–49.6)**	1.4
≥65 yrs, Asian	286	37.9	(31.1–45.2)**	1.4
≥65 yrs, other	98	63.2	(50.5–74.3)	1.2
**Tetanus vaccination including pertussis vaccine, past 6 yrs** §§				
19–64 yrs, total	17,480	12.5	(11.8–13.2)	4.3¶
19–64 yrs, white	9,482	13.8	(12.9–14.7)	4.7¶
19–64 yrs, black	2,784	11.0	(9.5–12.6)**	3.6¶
19–64 yrs, Hispanic	3,558	7.7	(6.6–8.9)**	2.9¶
19–64 yrs, Asian	1,250	11.7	(9.4–14.5)	2.5
19–64 yrs, other	406	19.7	(15.0–25.5)	11.3¶
19–64 yrs, living with an infant aged <1 yr	700	21.5	(17.9–25.6)	10.9¶
19–64 yrs, not living with an infant aged <1 yr	16,802	12.1	(11.4–12.8)	4.0¶
**Hepatitis A vaccination (≥2 doses), ever** ¶¶				
19–49 yrs, total	14,893	12.5	(11.8–13.3)	1.8¶
19–49 yrs, white	7,951	12.3	(11.3–13.2)	1.9¶
19–49 yrs, black	2,260	11.2	(9.4–13.2)	0.9
19–49 yrs, Hispanic	3,276	11.3	(9.8–12.9)	0.9
19–49 yrs, Asian	1,049	19.1	(15.7–23.0)**	3.8
19–49 yrs, other	357	21.1	(16.1–27.1)**	4.6
19–49 yrs, had traveled outside the United States to countries other than Japan, Australia, New Zealand, Canada, or the countries of Europe since 1995	5,361	20.1	(18.8–21.5)	3.5¶
19–49 yrs, had not traveled outside the United States to countries other than Japan, Australia, New Zealand, Canada, or the countries of Europe since 1995	9,505	8.4	(7.6–9.2)	0.9
19–49 yrs, with chronic liver conditions, overall	136	17.1	(10.9–25.7)	−2.7
**Hepatitis B vaccination (≥3 doses), ever** ***				
19–49 yrs, total	15,568	35.9	(34.9–36.9)	2.1¶
19–49 yrs, white	8,256	37.8	(36.5–39.2)	2.2
19–49 yrs, black	2,349	33.0	(30.7–35.3)**	−1.5
19–49 yrs, Hispanic	3,429	28.9	(27.1–30.9)**	3.6¶
19–49 yrs, Asian	1,144	40.7	(36.8–44.6)	3.5
19–49 yrs, other	390	44.1	(38.5–49.9)	6.6
19–59 yrs, with diabetes, overall	1,224	26.9	(23.8–30.3)	4.2
≥60 yrs, with diabetes, overall	1,746	12.4	(10.8–14.3)	1.5
**Herpes zoster (shingles) vaccination, ever** †††				
≥60 yrs, total	9,278	15.8	(14.8–16.9)	1.4
≥60 yrs, white	6,531	17.6	(16.4–18.9)	1.0
≥60 yrs, black	1,204	7.9	(6.2–9.9)**	3.4¶
≥60 yrs, Hispanic	978	8.0	(6.2–10.2)**	3.6¶
≥60 yrs, Asian	409	14.0	(10.4–18.6)**	1.3
≥60 yrs, other	156	12.0	(7.2–19.3)	3.8
**Human papillomavirus (HPV) vaccination among females (≥1 dose), ever** §§§				
19–21 yrs, total	718	43.1	(38.4–48.0)	14.9¶
22–26 yrs, total	1,459	21.5	(18.8–24.5)	5.0¶
19–26 yrs, total	2,177	29.5	(27.0–32.1)	8.8¶
19–26 yrs, white	1,083	32.5	(29.1–36.1)	10.1¶
19–26 yrs, black	388	28.3	(23.3–33.9)	7.9
19–26 yrs, Hispanic	480	20.2	(16.3–24.8)**	5.1
19–26 yrs, Asian	153	22.3	(16.0–30.2)	0.3
19–26 yrs, other	73	39.0	(25.6–54.3)	22.5
**HPV vaccination among males (≥1 dose), ever** §§§				
19–26 yrs, total	1,833	2.1	(1.4–3.2)	1.5¶
19–21 yrs, total	601	2.8	(1.6–4.9)	2.5¶
22–26 yrs, total	1,232	1.7	(0.9–3.2)	0.9

**Abbreviation:** CI = confidence interval.

* Adults were considered at high risk for pneumococcal disease if they had ever been told by a doctor or other health professional that they had diabetes, emphysema, coronary heart disease, angina, heart attack, or other heart condition; had a diagnosis of cancer during the previous 12 months (excluding nonmelanoma skin cancer); had ever been told by a doctor or other health professional that they had lymphoma, leukemia, or blood cancer; had been told by a doctor or other health professional that they had chronic bronchitis or weak or failing kidneys during the preceding 12 months; had an asthma episode or attack during the preceding 12 months; or were current smokers. Information on high-risk status for hepatitis B or A was not collected in 2011.

† Race/ethnicity was categorized as follows: Hispanic, black, white, Asian, and “other.” In this report, persons identified as Hispanic might be of any race. Persons identified as black, white, Asian, or other race are non-Hispanic. “Other” includes American Indian/Alaska Native and multiple race. The five racial/ethnic categories are mutually exclusive.

§ Respondents were asked if they had ever had a pneumonia shot.

¶ p<0.05 by t test for comparisons between 2011 and 2010 within each level of each characteristic.

** p<0.05 by t test for comparisons with whites as the reference.

†† Respondents were asked if they had received a tetanus shot in the past 10 years. Vaccinated respondents included adults who received tetanus-diphtheria toxoid (Td) during the past 10 years or tetanus, diphtheria, and acellular pertussis vaccine (Tdap) during 2005–2011.

§§ Respondents who had received a tetanus shot in the past 10 years were asked if their most recent shot was given in 2005 or later. Respondents who had received a tetanus shot since 2005 were asked if they were told that their most recent tetanus shot included the pertussis or whooping cough vaccine. Among 25,783 respondents aged 19–64 years, those without a “yes” or “no” classification for tetanus vaccination in the past 10 years (n = 1,118 [4.3%]) or for tetanus vaccination during 2005–2011 (n = 803 [3.1%]), and those who reported tetanus vaccination during 2005–2011 but were not told vaccine type by the provider (n = 5,501 [21.3%]) or did not know vaccine type (Td or Tdap) (n = 881 [3.4%]) were excluded, yielding a sample of 17,480 respondents aged 19–64 years for whom Tdap vaccination status could be assessed. Advisory Committee on Immunization Practices recommendations on use of Tdap in certain adults aged ≥65 years were published January 14, 2011.

¶¶ Respondents were asked if they had ever received the hepatitis A vaccine, and if yes, were asked how many shots were received.

*** Respondents were asked if they had ever received the hepatitis B vaccine, and if yes, if they had received ≥3 doses or <3 doses.

††† Respondents were asked if they had ever received a shingles vaccine.

§§§ Respondents were asked if they had ever received the HPV shot or cervical cancer vaccine.

**TABLE 2:** Type of tetanus vaccine received, and proportion that were tetanus, diphtheria, acellular pertussis (Tdap) vaccine, among adults aged 19–64 years who received a tetanus vaccination, by selected characteristics — National Health Interview Survey, United States, 2011

Characteristic	Type of vaccine received among those who received a tetanus vaccination during 2005–2011									Proportion Tdap of total tetanus vaccinations during 2005–2011*		

	No. in sample	Received Tdap		Received other tetanus vaccine		Doctor did not inform the patient		Could not recall vaccine type				
				
		%	(95% CI)	%	(95% CI)	%	(95% CI)	%	(95% CI)	No. in sample	%	(95% CI)
Adults aged 19–64 yrs	9,805	21.5	(20.4–22.6)	13.7	(12.7–14.7)	55.9	(54.5–57.3)	8.9	(8.2–9.7)	3,422	61.1	(58.8–63.3)
HCP aged 19–64 yrs†	1,230	37.3	(33.9–40.8)	18.5	(15.9–21.5)	38.8	(35.4–42.4)	5.4	(4.1–7.0)	695	66.8§	(62.2–71.0)
Non-HCP aged 19–64 yrs	8,565	19.3	(18.1–20.5)	13	(12.0–14.1)	58.3	(56.8–59.8)	9.4	(8.6–10.3)	2,723	59.7	(57.0–62.3)

**Abbreviations:** CI = confidence interval; HCP = health-care personnel.

* Calculated by dividing number of respondents who reported receiving Tdap by the sum of those who reported receiving Tdap and those who reported receiving other tetanus vaccination; respondents who reported that the doctor did not inform them of the vaccine type they received and those who could not recall the vaccine type were excluded.

† Adults were classified as HCP if they reported that they currently volunteer or work (full-time or part-time) in a hospital, medical clinic, doctor’s office, dentist’s office, or nursing home, or provided professional nursing care in the home.

§ p<0.05 by t test for comparisons between HCP and non-HCP aged 19–64 years.

**TABLE 3:** Estimated proportion of health-care personnel* who received selected vaccinations, by race/ethnicity† — National Health Interview Survey, United States, 2011

Characteristic	No. in sample	%	(95% CI)	Percentage point difference from 2010
**Tetanus vaccination including pertussis vaccine, past 6 yrs** §				
19–64 yrs, total	1,759	26.8	(24.2–29.5)	6.5¶
19–64 yrs, white	1,046	27.2	(24.1–30.6)	5.7
19–64 yrs, black	315	21.7	(16.4–28.1)**	7.7
19–64 yrs, Hispanic	217	30.1	(22.7–38.7)	16.3¶
19–64 yrs, Asian	146	27.8	(19.2–38.4)	0.9
19–64 yrs, other	35	31.2	(16.9–50.4)	—††
**Hepatitis B vaccination (≥3 doses), ever** §§				
≥19 yrs, total	2,564	63.8	(61.4–66.2)	0.6
≥19 yrs, white	1,581	65.1	(62.0–68.1)	1.3
≥19 yrs, black	432	57.1	(50.5–63.4)**	−1.7
≥19 yrs, Hispanic	314	59.4	(51.7–66.7)**	2.4
≥19 yrs, Asian	186	70.4	(61.6–77.8)**	−2.4
≥19 yrs, other	51	70.0	(50.9–84.0)	−0.2

**Abbreviation:** CI = confidence interval.

* Adults were classified as health-care personnel if they reported that they currently volunteer or work (full-time or part-time) in a hospital, medical clinic, doctor’s office, dentist’s office, or nursing home, or provided professional nursing care in the home.

† Race/ethnicity was categorized as follows: Hispanic, black, white, Asian, and “other.” In this report, persons identified as Hispanic might be of any race. Persons identified as black, white, Asian, or other race are non-Hispanic. “Other” includes American Indian/Alaska Native and multiple race. The five racial/ethnic categories are mutually exclusive.

§ Respondents who had received a tetanus shot in the past 10 years were asked if their most recent shot was given in 2005 or later. Respondents who had received a tetanus shot since 2005 were asked if they were told that their most recent tetanus shot included the pertussis or whooping cough vaccine. Among 2,439 health-care personnel aged 19–64 years, those without a “yes” or “no” classification for tetanus vaccination status in the past 10 years (n = 60 [2.5%]) or for tetanus vaccination status during 2005–2011 (n = 85 [3.5%]), and those who reported tetanus vaccination during 2005–2011 but were not told vaccine type by the provider (n = 463 [19.0%]) or did not know vaccine type (Td or Tdap) (n = 72 [3.0%]) were excluded, yielding a sample of 1,759 respondents aged 19–64 years for whom Tdap vaccination status could be assessed. Advisory Committee on Immunization Practices recommendations on use of Tdap in certain adults aged ≥65 years were published January 14, 2011.

¶ p<0.05 by t test for comparisons between 2011 and 2010 within each level of each characteristic.

** p<0.05 by t test for comparisons with whites as the reference.

†† Estimate is not reliable because of small sample size (n<30) or relative standard error (standard error/estimates) >0.3.

§§ Respondents were asked if they had ever received the hepatitis B vaccine, and if yes, if they had received ≥3 doses or <3 doses.

## Footnotes

* *Healthy People 2020* objectives and targets for immunization and infectious diseases are available at http://www.healthypeople.gov/2020/topicsobjectives2020/objectiveslist.aspx?topicid=23.

† Race/ethnicity was categorized as follows: Hispanic, black, white, Asian and “other.” In this report, persons identified as Hispanic might be of any race. Persons identified as black, white, Asian, or other race are non-Hispanic. “Other” includes American Indian/Alaska Native and multiple race. The five racial/ethnic categories are mutually exclusive.

§ Adults were considered at high risk for pneumococcal disease if they had ever been told by a doctor or other health professional that they had diabetes, emphysema, coronary heart disease, angina, heart attack, or other heart condition; had a diagnosis of cancer during the previous 12 months (excluding nonmelanoma skin cancer); had ever been told by a doctor or other health professional that they had lymphoma, leukemia, or blood cancer; had been told by a doctor or other health professional that they had chronic bronchitis or weak or failing kidneys during the preceding 12 months; had an asthma episode or attack during the preceding 12 months; or were current smokers. Information on high-risk status for hepatitis B or A was not collected in 2011.

¶ Additional information on NHIS methods is available at http://www.cdc.gov/nchs/nhis/methods.htm.

** In 2011, a single dose of Tdap was recommended for adults aged ≥65 years who have or who anticipate having close contact with an infant aged <1 year (e.g., grandparents, child-care providers, and health-care personnel) to reduce the risk for transmitting pertussis. Other adults aged ≥65 years may receive Tdap.

†† Additional information available at http://www.cdc.gov/mmwr/preview/mmwrhtml/mm6125a4.htm.

§§ Additional information available at http://www.cdc.gov/mmwr/preview/mmwrhtml/mm6140a4.htm?s_cid=mm6140a4_w.
